# Impact of donor specific antibodies on longitudinal lung function and baseline lung allograft dysfunction

**DOI:** 10.1016/j.healun.2025.06.012

**Published:** 2025-06-26

**Authors:** Muhtadi Alnababteh, Junfeng Sun, Rohan Meda, Lucia Ponor, Pali Shah, Joby Mathew, Hyesik Kong, Ananth Charya, RN Helen Luikart, Shambhu Aryal, Steven D. Nathan, Jonathan B. Orens, Kiran K. Khush, Moon Jang, Sean Agbor-Enoh, Michael B. Keller

**Affiliations:** a Laborarory of Applied Precision Omics (APO), National Heart, Lung and Blood Institute (NHLBI), National Institutes of Health, Bethesda, MD; b Laboratory of Transplantation Genomics, National Heart, Lung and Blood Institute (NHLBI), National Institutes of Health, Bethesda, MD; c Genomic Research Alliance for Transplantation (GRAfT); d Pulmonary and Critical Care Medicine, Johns Hopkins Hospital, Baltimore MD; e Division of Hospital Medicine, Johns Hopkins Bayview Medical Center, Baltimore, MD; f Critical Care Medicine Department, Clinical Center, National Institutes of Health, Bethesda, MD; g Inova Fairfax Hospital, Falls Church, VA; h Division of Pulmonary and Critical Care Medicine, University of Maryland Medical Center, Baltimore MD; i Genome Transplant Genomics (GTD); j Division of Cardiovascular Medicine and Stanford University School of Medicine, Palo Alto, CA; k Department of Pathology, Stanford University School of Medicine, Palo Alto, CA.

**Keywords:** BLAD, DSA, Lung Transplant

## Abstract

**BACKGROUND:**

Lung transplantation offers life-saving benefits for patients with end-stage lung disease, however, long-term outcomes remain poor, with a median survival of 6.5 years. Identifying patients at risk for poor post-transplant lung function is crucial for improving outcomes. While peri-operative and demographic factors have previously been studied, the impact of donor-specific antibodies (DSA) on longitudinal post-transplant lung function remains unclear. This study examines the effects of DSA on post-transplant lung function and the risk of baseline lung allograft dysfunction (BLAD).

**RESEARCH QUESTION:**

Is DSA development linked to worse longitudinal lung function, higher BLAD rates, and poorer survival compared to DSA-negative patients regardless of the development of clinical AMR?

**METHODS:**

This study included lung transplant recipients from two prospective cohort studies, comparing DSA+ and DSA- patients. All participants underwent serial surveillance and clinically-indicated bronchoscopy, pulmonary function tests, and DSA testing. Statistical analysis included linear mixed models for longitudinal lung function data, multivariable logistic regression for BLAD, and survival analysis using Cox Proportional Hazard models.

**RESULTS:**

We analyzed 213 patients with a median follow-up of 48.1 months. Among them, 50.7% developed DSA. DSA+ patients showed significantly lower rates of post-transplant spirometric improvement compared to DSA- patients (p=0.008 for %FVC; p=0.02 for %FEV1). After DSA detection, there was a significant decrease in the slopes of %FVC and %FEV1 (p=0.0008 and p=0.0006, respectively). DSA+ patients had a higher risk of developing BLAD (OR 2.14, 95% CI [1.45, 3.17], p=0.0001). Additionally, DSA+ patients had a higher risk of death (HR 2.98, 95% CI [1.79, 4.99], p < 0.0001). These findings were consistent even when excluding patients with clinical antibody-mediated rejection (AMR).

**INTERPRETATION:**

Our study demonstrates that DSA development significantly impairs post-transplant lung function and increases the risk of BLAD even in the absence of clinical AMR. These findings suggest that DSA may serve as a biomarker of BLAD, and could potentially aid in risk stratification following lung transplantation.

## Introduction

Although lung transplantation is a life-saving procedure for patients with end-stage lung disease, long-term outcomes remain poor, with a median survival of 6.5 years.^1,2^ The goal of lung transplantation is to improve survival and quality of life through the restoration of lung function. However, some patients never achieve normal predicted lung function.^3,4^ Lower peak lung function after transplantation is associated with increased mortality.^5^ It is therefore essential to identify patients at risk for poor post-transplant lung function in order to properly risk stratify these patients and identify modifiable risk factors.

The majority of risk factors identified for poor baseline lung function have centered around peri-operative or demographic risk factors, including primary graft dysfunction (PGD), recipient obesity and donor age.^5^ However, while the development of donor specific antibodies (DSA) has been associated with a variety of adverse post-transplant outcomes, including antibody-mediated rejection (AMR), Chronic Lung Allograft Dysfunction (CLAD), and increased mortality,^6–8^ the impact of DSA on longitudinal lung function and baseline lung allograft dysfunction (BLAD) remains unclear. This study aims to address these knowledge gaps by examining the longitudinal effects of DSA on post-transplant lung function and the risk of BLAD. Additionally, we sought to explore the association between DSA and specific CLAD phenotypes. We hypothesized that patients who develop DSA will demonstrate worse lung function trajectories and higher rates of BLAD. By examining the impact of DSA on various aspects of lung allograft function and long-term outcomes, these findings may have important implications for risk stratification, monitoring strategies, and therapeutic interventions in this patient population.

## Methods

We performed a multicenter, observational analysis that included lung transplant recipients > 18 years of age enrolled in two prospective cohort studies. The first study, Genome Transplant Dynamics (GTD) (NCT01985412), enrolled lung transplant recipients at a single center (Stanford University Hospital) between December 1, 2010, and December 31, 2012 with follow up until May 1, 2019. The second study, Genome Research Alliance for Transplantation (GRAfT) (NCT0243070), began enrollment in 2015 and is ongoing at three centers (Inova Fairfax Hospital, Johns Hopkins Hospital, and University of Maryland Medical Center). All patients underwent routine post-transplant monitoring with regularly scheduled clinic visits, lab draws, pulmonary function testing (PFT) and surveillance bronchoscopy with bronchoalveolar lavage (BAL) and transbronchial biopsy (TBBx). We excluded patients with insufficient PFT data. For analyses incorporating the endpoint of BLAD, we excluded single lung transplants. All other analyses, including those using CLAD as an endpoint, we included both single and double lung transplant. This study was approved by the Institutional Review Board at each participating center and the National Heart, Lung, and Blood Institute.

### DSA testing and measurement

The participating centers performed surveillance HLA testing prior to transplantation and on post-transplant days 7 and 14 and months 1, 3, 6, 9, 12, 18, and 24 (coincident with scheduled surveillance bronchoscopy). Patients underwent additional DSA testing for clinical signs or symptoms of allograft dysfunction. DSAs were detected at each center by single antigen bead testing using the LABScreen Single Antigen Bead assay (One Lambda) and designated as either positive or negative. A positive test was defined as a mean fluorescence intensity (MFI) ≥ 1000 on 1 occasion or an MFI between 500 and 1000 on 2 serial occasions as this was the definition of positive of DSA by centers. Details on the DSA treatment practices at participating centers are included in supplemental material.

### Outcomes

The primary outcomes of this study were differences in longitudinal post-transplant lung function trajectory and the incidence of BLAD. Secondary outcomes included CLAD and death. BLAD was defined as failure to achieve ≥ 80% predicted for both forced expiratory volume in one second (%FEV1) and forced vital capacity after lung transplant, on 2 consecutive measurements at least 3 months apart without a > 20% increase in %FEV1 in the 3 months prior to their last recorded PFT values. The time of BLAD was defined as the time of baseline lung function in the cohort that failed to reach normal lung function.^5^ For the BLAD analysis, only cases where DSA occurred before BLAD diagnosis or within one year after BLAD diagnosis were included (in order to capture the possibility that DSA developing in a window after BLAD diagnosis continues to inhibit a rise in lung function). CLAD was defined by International Society for Heart and Lung Transplantation (ISHLT) criteria as a sustained decrease of ≥ 20% %FEV1 from baseline value at least 3 months post-transplant and taken at least 3 weeks apart.^9^

### Statistical analysis

Continuous variables were described using mean (standard deviation) or median (interquartile range), and categorical variables were summarized using counts (percentage). Nonparametric tests were used when indicated. Univariate analyses were conducted to compare primary and secondary endpoints using χ^2^ or Fisher exact test for categorical variables and t tests for continuous variables. Longitudinal lung function data were modeled using linear mixed models with random intercepts and slopes for each patient to account for within-patient correlation. Data were log_10_-transformed to linearize the trend over time. First, we compared the overall trends between DSA+ and DSA- groups. Next, a change point was introduced for each DSA+ patient at the time of DSA detection. Standard residual diagnostics were used to check for model assumptions. Multivariable logistic regression was used to assess the association of DSA with BLAD and DSA with RAS vs BOS phenotypes, adjusting for recipient age and BMI, site, native lung disease, development of PGD grade 3 at 72 h and single vs double lung transplant (single vs double lung transplant was not included as a covariate in the BLAD analysis because all patients adjudicated for BLAD were double lung transplant patients). Generalized estimating equations (GEE) were used to account for clustering of patients within each site. For survival analysis, a Cox Proportional Hazard (CoxPH) model was used for death. For CLAD, we used a cause specific CoxPH model with death as a competing risk. DSA was modeled as a time-dependent variable. Proportional hazard assumptions were checked. SAS version 9.4 was used unless noted otherwise. A p value ≤ 0.05 was considered statistically significant.

## Results

**Figure 1:**
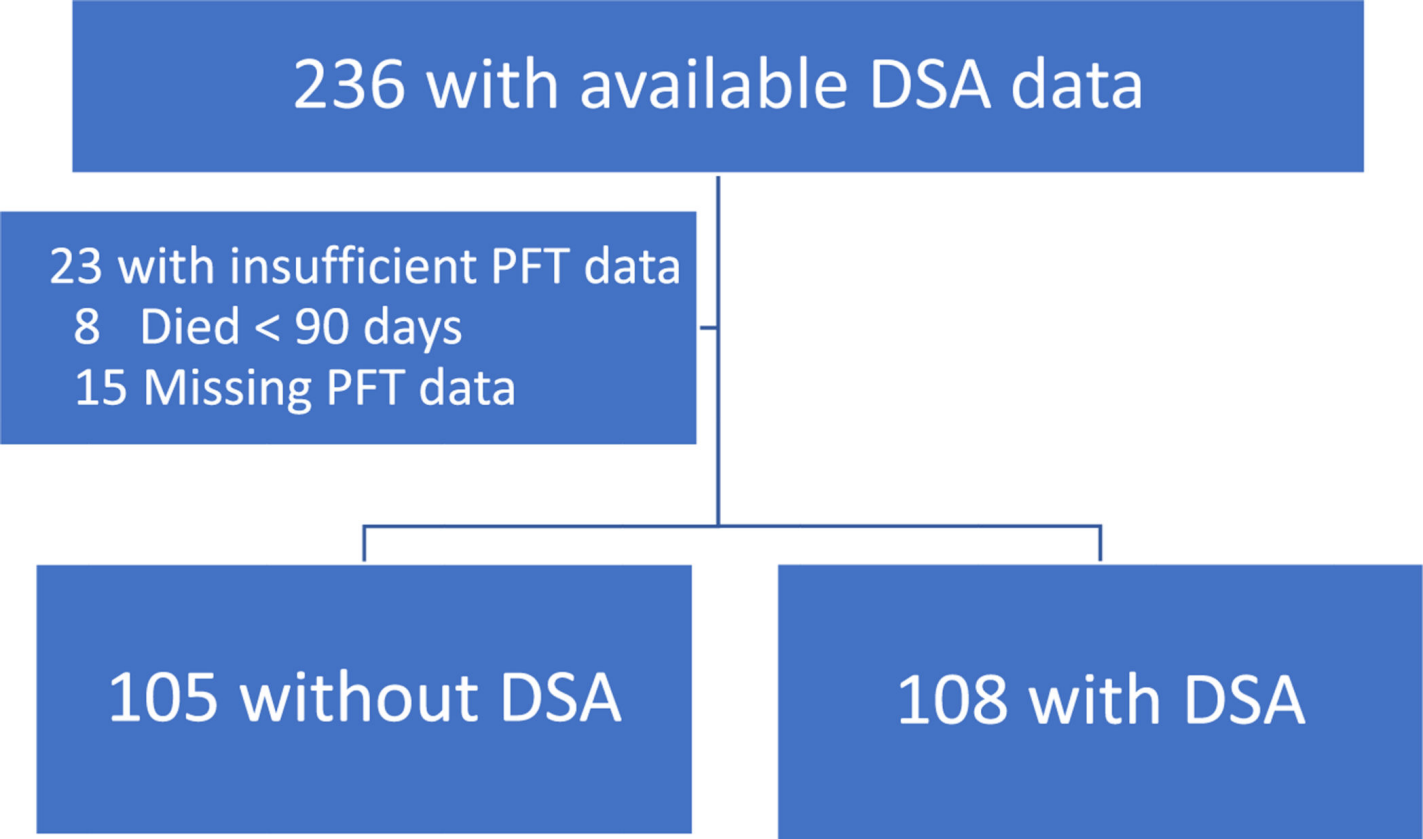
Flow Chart of Patient Enrollment and Outcomes. This figure illustrates the flow chart of patient enrollment in the study. 236 patients were included from two cohorts: GRAFT and GTD. Some patients were excluded due to incomplete data. The final analysis included patients categorized into two groups based on the presence of donor-specific antibodies (DSA): DSA+ and DSA-.

Two hundred and thirty six subjects had available DSA data in the two cohort studies, GRAfT and GTD. Of these, 23 patients had insufficient PFT data; 8 died within 90 days of transplant, 15 had incomplete data leaving 213 patients included in the final analysis (Figure 1). The median (IQR) length of post-transplant follow-up was: 48.1 (33.9 – 64.2) (min, max): (1.3, 89.3) months. The median age (IQR) of the cohort was 58.0 (44.7 – 64.2) years. Of the participants, 178 (83.6%) patients were White, and 106 (49.8%) were female (Table 1). One hundred and eight subjects (50.7%) developed DSA, with the median (IQR) day of detection being 55.5 (24−369). Among those who developed DSA, 80 (74.1%) had HLA-DQ antibodies, either alone or in combination with other antibodies. Additionally, 70 (64.8%) of the DSA+ patients received treatment for DSA, and 42 (40.4%) cleared their DSA antibodies (Table 2).

### Differences in longitudinal lung function between DSA+ vs DSA- patients

**Figure 2:**
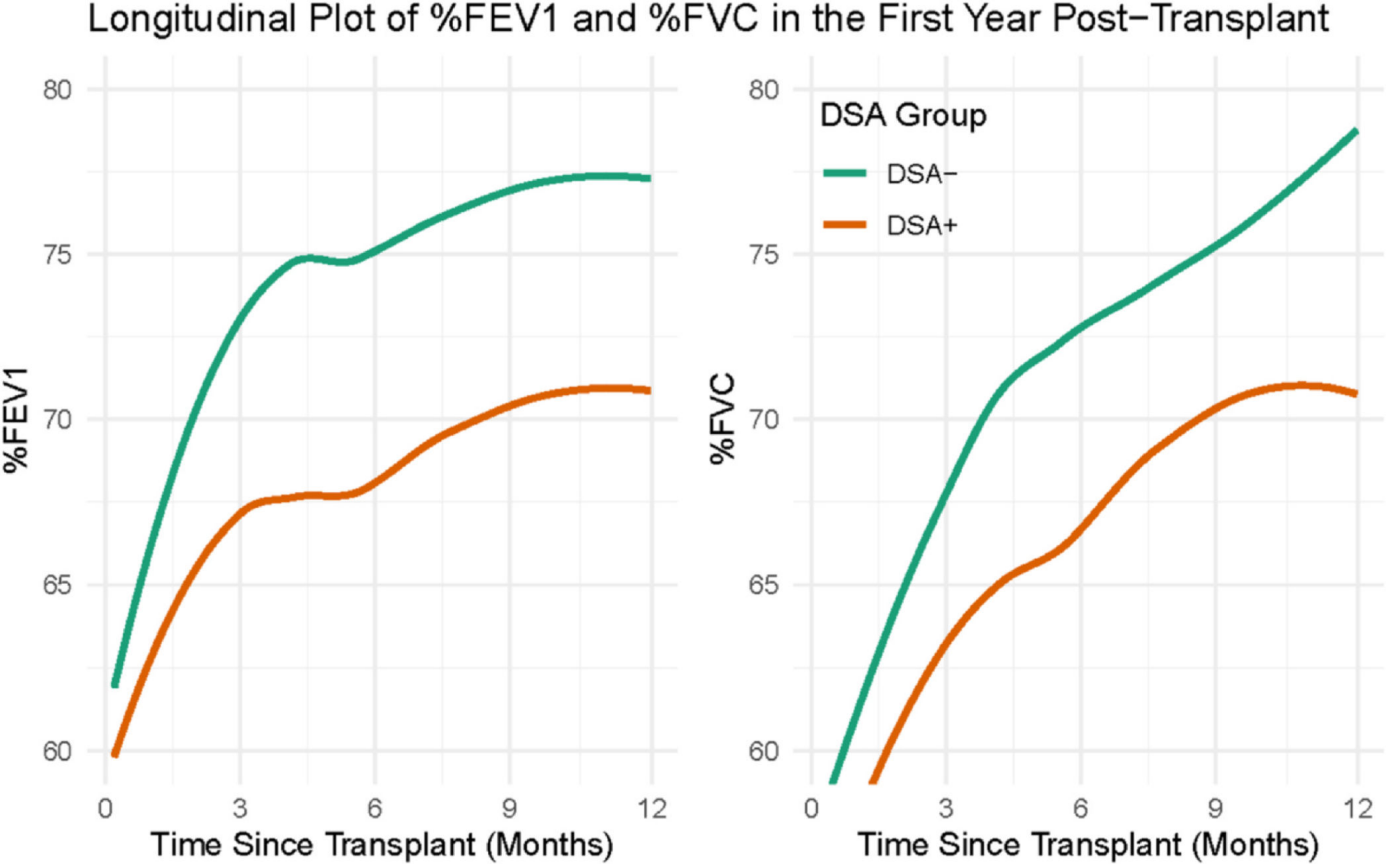
Longitudinal Plot of %FEV1 and %FVC in the First Year Post-Transplant. This figure shows the longitudinal trends of % predicted forced expiratory volume in the first second (%FEV1) and % predicted forced vital capacity (%FVC) in lung transplant recipients over the first-year post-transplant. The y-axes represent %FEV1 (left) and %FVC (right), while the x-axes represent time since transplant in months. The plots compare two groups based on the presence of donor-specific antibodies (DSA): DSA- (green line) and DSA+ (orange line). The DSA- group demonstrates higher and more consistent improvements in both %FEV1 and %FVC compared to the DSA+ group throughout the first-year post-transplant. The DSA+ group shows a slower and lower improvement in lung function. The plots were generated using the LOESS (Locally Estimated Scatterplot Smoothing) method to fit smooth curves to the data points. DSA, donor-specific antibodies; %FEV1, percent predicted forced expiratory volume in the first second; %FVC, percent predicted forced vital capacity.

DSA+ patients demonstrated differences in longitudinal lung function compared to DSA- patients (Figure 2). In the analysis comparing overall trends, days post transplant were log_10_-transformed to linearize the relationship. Both groups showed improvement of their %FEV1 and %FVC, however, DSA+ patients exhibited a significantly lower rate of post-transplant spirometric improvement compared to DSA negative patients (−5.57% (2.11), p=0.008 for %FVC; −6.75% (2.81), p=0.02 for %FEV1). Here, the slope is the change in %FEV1 or % FVC per 10-fold change in days post-transplant (e.g. from day 1 to day 10, day 10 to day 100, etc). To further understand the impact of DSA development on lung function, we introduced change points for each DSA+ patient at the time of DSA detection. Before the appearance of DSA, the slopes for % FVC and %FEV1 in DSA+ patients were not significantly different from those observed in DSA- patients (2.64% (2.36), p=0.26 for %FVC; 1.97% (3.20), p=0.54 for %FEV1); however, after the detection of DSA+, there was a significant decrease in the slopes for both %FVC and %FEV1, (−46.53% (13.11), p=0.0008 for %FVC; −42.93% (11.86), p=0.0006 for %FEV1). These results remained consistent when comparing only De Novo DSA+ patients to DSA- patients (−69.35% (19.89), p=0.001 for %FVC; −64.42% (17.91), p=0.0007 for %FEV1), as well as comparing DSA+ patients without evidence of clinical AMR to DSA- patients(−74.74% (32.72), p=0.03 for %FVC; −73.11% (30.46), p=0.02 for %FEV1). There was no significant difference in longitudinal FEV1:FVC ratios between DSA+ and DSA- patients (Table 3). Trends in post-transplant pulmonary function did not differ between DSA+ patients who received treatment compared to DSA+ patients who did not receive treatment (−40.35% (31.65), p=0.20 for %FVC; −28.50% (29.57), p=0.34 for %FEV1). They also did not differ between DQ DSA and non-DQ DSA (−37.61% (30.28), p=0.21 for %FVC; −27.52% (28.12), p=0.33 for %FEV1).

### Association of DSA with the development of baseline lung allograft dysfunction

Overall, 60 (39%) subjects developed BLAD, with a median (IQR) time to BLAD of 246 (105−418) days. In multivariable logistic regression analysis, DSA+ patients had a higher risk for developing BLAD compared to DSA- patients (OR (CI) 2.14 (1.45, 3.17), p =0.0001) but had a similar median time to reach baseline function 375 (297) vs 381 (329) days, p= 0.74 (Table 4). These results remained consistent when considering DSA+ patients without evidence of clinical AMR compared with DSA- patients without evidence of clinical AMR, OR (CI) 2.37 (1.55, 3.64), p < 0.0001; comparing only De Novo DSA+ patients to DSA- patients (1.92 (1.42, 2.57), p < 0.0001), and comparing patients with DSA by 30 days to those with DSA > 30 days (1.34 (1.21, 1.48), p < 0.0001). DSA+ patients who received treatment had similar rates of BLAD compared to DSA+ patients who did not receive treatment (p=0.32). There was no statistical differences in risk of BLAD between DQ DSA compared to non-DQ DSA patients (p=0.31).

### Association of DSA with CLAD and survival

Sixty-seven (31%) patients developed CLAD over the study period, including 43 (64%) with BOS and 24 (36%) with RAS. In multivariable Cox regression analysis, DSA+ subjects had a higher risk of CLAD development compared to DSA- subjects (HR(CI), 2.09 (1.28, 3.40), p=0.003). Further, there was a distinct distribution between RAS and BOS phenotypes, with DSA+ subjects having higher odds of developing RAS compared to BOS (OR(CI) 5.28 (2.08, 13.43), p=0.0005). DSA+ subjects had a higher risk of death compared to DSA- patients (HR(CI) 2.98 (1.79, 4.99), p < 0.0001). These results remained consistent when considering DSA+ patients without evidence of clinical AMR compared with DSA- patients (HR (CI): 2.65 (1.32, 5.32), p=0.006 for death); and comparing only De Novo DSA+ patients to DSA- patients (HR (CI): 3.62 (2.14, 6.14), p < 0.0001).

## Discussion

This study provides novel insights into the impact of DSA on lung transplant outcomes, offering the first comprehensive analysis of DSA effects on longitudinal allograft function and BLAD in a multicenter prospective cohort study. Our results reveal significant associations between DSA positivity and a variety adverse outcomes, including lower post-transplant lung function trends, higher risk of developing BLAD and CLAD, and increased mortality. These findings may have clinical implications for the risk stratification and potential management of DSA+ patients.

Lung function is expected to gradually improve post-transplantation; however, our study suggests that the presence of DSA may mitigate this improvement rather than simply causing acute declines in lung function. Patients with DSA exhibited a lower upward trend in %FEV1 and %FVC, which occurred *after* the development of DSA. This contrasts with previous studies that primarily emphasize acute *declines* in lung function rather than the lack of expected improvement in post-transplant lung function.^10^ These findings may have significant clinical implications. While the traditional focus has been on the immediate worsening of lung function as a clinically relevant outcome, our results suggest that the lack of improvement in lung function improvement may be equally consequential in patients developing DSA in the initial months after transplantation. Notably, these effects were observed even in patients who never developed clinical AMR, indicating that the decline in lung function in DSA+ subjects cannot be solely attributed to clinical AMR. This highlights the potential subclinical impact of DSA on allograft function. Further, the lack of significant improvement in lung function among DSA+ patients who received treatment compared to those who did not suggests that current treatment approaches may be insufficient or initiated too late in the disease process. Our findings suggest further examination and potential reconsideration into the clinical practice of treating DSA only when clinical AMR is diagnosed or when a decline in lung function is observed,^11^ as previous studies have shown that preemptive DSA treatment may result in improved long-term outcomes, including reduced rates of CLAD and death.^12^ The observed donor-specific antibody (DSA) prevalence in our cohort (50.7%) is consistent with previously reported rates in lung transplant recipients undergoing routine surveillance, which range from 30% to 50%.^8,11^ In contrast, centers using for-cause testing protocols tend to report lower DSA rates (17%), likely due to underdetection of subclinical alloimmune responses.^10^

The association between DSA positivity and a higher risk of developing BLAD is a novel finding in our study. BLAD, defined as the failure to achieve normal baseline lung function post-transplant, is becoming increasingly recognized as a critical determinant of long-term allograft health.^5^ Interestingly, this correlation persists even among patients who are DSA-positive but do not exhibit clinical signs of AMR, suggesting that DSAs may be influencing the onset of BLAD through pathways that are not immediately identifiable by current clinical AMR diagnostic criteria. The biological rationale for this connection is reinforced by the observation that DSA often emerge shortly after the transplant, which could interfere with the patient’s ability to achieve normal baseline lung function. In a prior study by Wang et al., which included only pre-transplant DSA, the proportion of double lung transplant recipients with BLAD did not differ by DSA status (37% vs. 46%; P = 0.419).^13^ While our cohort included some patients with pre-transplant DSA, the vast majority developed DSA post-transplant, which may account for the differences in observed associations in our study from Wang et al. These findings suggest that early identification and monitoring of post-transplant DSA may be important in understanding and mitigating the development of BLAD.

Consistent with prior studies, our study also demonstrated a strong association between DSA positivity and the development of CLAD. Echoing these earlier findings, our study indicates that the presence of DSA is associated with patterns of lung disease that are restrictive in nature.^8^ This is supported by the observation that there was no notable difference in the ratio of FEV1 to FVC between patients with and without DSAs, and that patients with DSAs were found to have a greater likelihood of developing RAS as opposed to BOS. The varying influence of DSA on the emergence of RAS versus BOS points to the possibility of different underlying pathophysiological mechanisms, highlighting the need for further investigation into the mechanisms by which DSA contribute to RAS development, which may provide valuable insights into potential therapeutic targets. Additionally, the progression from BLAD to CLAD introduces an intriguing concept, as patients with lower baseline predicted lung function must still experience a 20% decline in lung function to warrant a diagnosis of CLAD. Patients with severe BLAD may not have the physiologic reserve to tolerate this persistent spirometric decline over the time period required for a diagnosis of CLAD before experiencing mortality. In this manner, we may be underestimating the true incidence of CLAD in patients with BLAD. Further research is warranted to evaluate whether an adjusted CLAD threshold for BLAD patients more accurately reflects the progression of lung dysfunction in this population.

This study has several limitations that should be acknowledged. First, the observational nature of the study may introduce selection bias, and also precludes the establishment of causality. Second, the heterogeneity in treatment regimens for DSA+ patients may confound the interpretation of the impact of treatment on lung function outcomes. Finally, the study did not assess the impact of DSA characteristics (e.g., titer, specificity) on outcomes, which could provide additional insights into risk stratification. In addition, data on potential contributors to pulmonary function decline such as airway complications or chest wall pathology were not routinely captured and may represent unmeasured confounders. Future prospective studies are needed to validate these findings and explore the potential benefits of standardized DSA monitoring and targeted therapeutic interventions. Understanding the mechanisms of DSA-mediated lung allograft injury will be crucial for developing comprehensive strategies to improve long-term outcomes in lung transplant recipients.

In conclusion, we demonstrate that the development of DSA is associated with poor post-transplant longitudinal lung function and the development of BLAD. Our findings suggest a potential value for routine DSA monitoring in the post-transplant period and that a more proactive approach to DSA treatment may be warranted, even in the absence of overt AMR or acute lung allograft dysfunction.

## Supplementary Material

1

## Figures and Tables

**Table 1:** Demographics, Clinical Characteristics, and Outcomes of Lung Transplant Recipients by DSA Status

	DSA - (N=105)	DSA+ (N=108)	Overall (N=213)

AGE			
Median (Q1-Q3)	60.1 (45.6–65.2)	54.9 (43.8–63.3)	58.0 (44.7–64.2)
RACE			
White	88 (83.8%)	90 (83.3%)	178 (83.6%)
Black	11 (10.5%)	16 (14.8%)	27 (12.7%)
Other	6 (5.7%)	2 (1.9%)	8 (3.8%)
BMI^†^			
Median (Q1-Q3)	25.0 (21.7–28.2)	25.9 (22.0–28.3)	25.3 (21.8–28.2)
Native Lung Disease			
COPD	19 (18.1%)	23 (21.3%)	42 (19.7%)
CF	19 (18.1%)	19 (17.6%)	38 (17.8%)
ILD	49 (46.7%)	50 (46.3%)	99 (46.5%)
PAH	2 (1.9%)	4 (3.7%)	6 (2.8%)
Sarcoidosis	3 (2.9%)	5 (4.6%)	8 (3.8%)
Other	13 (12.4%)	7 (6.5%)	20 (9.4%)
Lung Allocation Score			
Median (Q1-Q3)	42.8 (35.9–51.5)	40.8 (35.7–55.0)	41.4 (35.8–54.0)
Sex^†^			
Male	63 (60.0%)	44 (40.7%)	107 (50.2%)
Female	42 (40.0%)	64 (59.3%)	106 (49.8%)
Type of Lung Transplant			
Double	80 (76.2%)	84 (77.8%)	164 (77.0%)
Single	25 (23.8%)	24 (22.2%)	49 (23.0%)
Outcomes			
BLAD^†^	39 (37.5%)	56 (51.9%)	95 (44.8%)
PGD3	14 (13.7%)	20 (18.5%)	34 (16.2%)
CLAD^†^	24 (22.9%)	43 (39.8%)	67 (31.5%)
Type of CLAD^†^			
BOS	20 (83.3%)	23 (53.5%)	43 (64.2%)
RAS	4 (16.7%)	20 (46.5%)	24 (35.8%)
Death^†^	25 (23.8%)	45 (41.7%)	70 (32.9%)

Statistically significant differences between the DSA+ and DSA- groups were observed for sex, BLAD, CLAD, type of CLAD, BMI, and death. All other variables were not significantly different between groups (p > 0.05).

DSA, donor-specific antibodies; N, number; Q1, first quartile; Q3, third quartile; BMI, body mass index; COPD, chronic obstructive pulmonary disease; CF, cystic fibrosis; ILD, interstitial lung disease; PAH, pulmonary arterial hypertension; BLAD, Baseline Lung Allograft Dysfunction; PGD3, primary graft dysfunction grade 3; CLAD, Chronic Lung Allograft dysfunction; BOS, Bronchiolitis Obliterans Syndrome; RAS, Restrictive Allograft Syndrome.

**Table 2:** Characteristics of Donor Specific Antibodies and Treatment

	N=108

Time of DSA Development	
Preformed before Transplant	2 (1.9%)
Detected D0-D14	29 (26.9%)
De Novo > D14	75 (69.4%)
Preformed +De Novo	2 (1.9%)
DSA Specification	
A,B,C	11 (10.2%)
DQ	59 (54.6%)
DP/DR	12 (11.1%)
DQ+DP/DR	8 (7.4%)
DQ+CLASS I	6 (5.6%)
DQ+CLASS 1+DP/DR	7 (6.5%)
OTHER COMBINATIONS	4 (3.7%)
NON-HLA	1 (0.9%)
Received Treatment	70 (64.8%)
Plasma Exchange	44 (41.1%)
Rituximab	41 (38.3%)
IVIG	48 (44.9%)
Time of Treatment (Days Post Transplant)	
Median (Q1-Q3)	114 (38.8–353)
DSA cleared	
	42 (40.0%)
Maximum Strength	
MFI < 2000	42 (38.9%)
MFI 2000–10000	29 (26.8%)
MFI > 10000	29 (26.8%)
Missing	8 (7.4%)

**Table 3:** Estimated Slopes and p-values for %FVC and %FEV1 in DSA- and DSA+ Patients Before and After DSA+ Detection

	DSA- vs DSA+	DSA+ Before Change Point vs DSA-	DSA+ After vs Before Change Point

%FVC	12.18 (1.31), p < 0.0001	2.64 (2.36), p=0.26	−46.53 (13.11), p=0.0008
%FEV1	7.76 (1.81), p < 0.0001	1.97 (3.20), p=0.54	−42.93 (11.86), p=0.0006

This table presents the estimated slopes (standard error) and p-values for %FVC and %FEV1 from linear mixed models with random intercepts and slopes within each subject. The models use log_10_(days post transplant). The analysis compares DSA- (DSA negative) and DSA+ (DSA positive) patients before DSA detection, and DSA+ patients before and after DSA detection.

**DSA-:** Slopes (SE) and p-values for DSA- patients.

**DSA+ Before Change Point vs DSA-:** Difference in slopes (SE) and p-values between DSA+ patients before DSA+ detection and DSA- patients.

**DSA+ After vs Before Change Point:** Difference in slopes (SE) and p-values between before and after DSA+ detection for DSA+ patients. Slopes for DSA+ patients before the detection of DSA+ vs. DSA- patients were not significantly different. After the DSA+ detection, DSA+ patients had significant reductions in slopes for both %FVC and %FEV1 compared to themselves before DSA+ detection.

DSA, donor-specific antibodies; %FEV1, percent predicted forced expiratory volume in the first second; %FVC, percent predicted forced vital capacity.

**Table 4:** Multivariate Analysis of BLAD, CLAD, RAS, and Death in Lung Transplant Recipients, by presence of DSAs and AMR

	BLAD		CLAD		RAS		Death	
Models	OR (95% CI)	p	HR (95% CI)	p	OR (95% CI)	p	HR (95% CI)	p

DSA+ vs DSA- (Multivariate)	2.14 (1.45,3.17)	0.0001	2.09 (1.28, 3.40)	0.003	5.28 (2.08, 13.43)	0.0005	2.98 (1.79, 4.99)	< 0.0001
DSA+ without AMR vs DSA-	2.37 (1.55,3.64)	< 0.0001	1.27 (0.62, 2.61)	0.51	6.73 (2.44, 18.58)	0.0002	2.65 (1.32, 5.32)	0.006

This table presents the results of multivariate analyses examining the OR and HR with 95% CI for various outcomes in lung transplant recipients. The outcomes analyzed BLAD, CLAD, RAS, and death. The analyses are adjusted for multiple covariates in Model 2 and include a subgroup analysis for patients with DSA+ without AMR.

OR, odds ratio; HR, hazard ratio; CI, confidence interval; BLAD, Baseline Lung Allograft Dysfunction; CLAD, Chronic Lung Allograft Dysfunction; RAS, Restrictive Allograft Syndrome; DSA, donor-specific antibodies; AMR, antibody-mediated rejection.

## Appendix A. Supporting information

Supplemental data associated with this article can be found in the online version at doi:10.1016/j.healun.2025.06.012.

## Abbreviations:

ACR: Acute Cellular Rejection
AMR: Antibody Mediated Rejection
AR: Acute Rejection
BLAD: Baseline Lung Allograft Dysfunction
BAL: Bronchoalveolar Lavage
CLAD: Chronic Lung Allograft Dysfunction
dd-cfDNA: donor-derived cell-free DNA
DSA: Donor Specific Antibody
FEV1: Forced Expiratory Volume in One Second
FVC: Forced Vital Capacity
PGD: Primary Graft Dysfunction
PFT: Pulmonary Function Testing
TBBx: Transbronchial Biopsy
