# Supplementary material for: Impact of donor specific antibodies on longitudinal lung function and baseline lung allograft dysfunction
Source: J Heart Lung Transplant. Author manuscript; Available in PMC 2026 Jun 10. (PMC13251798; doi:10.1016/j.healun.2025.06.012)
Supplement: 1 [file NIHMS2168615-supplement-1.docx]

**Data Supplement**

**Impact of Donor Specific Antibodies on Longitudinal Lung Function and Baseline Lung Allograft Dysfunction**

Muhtadi Alnababteh, MD^1,6^, Junfeng Sun, Ph.D^7^, Rohan Meda^2^, Lucia Ponor, MD^3,5^, Pali Shah, MD^3,4^_,_ Joby Matthews, DPT^3,4^, Hyesik Kong, Ph.D^1,2,3^, Ananth Charya, MD^8^, Helen Luikart, RN^9,10,11^, Shambhu Aryal, MD^3,7^, Steven D. Nathan, MD^3,7^, Jonathan B. Orens, MD^3,4^, Kiran K. Khush, MD^9,10^, Moon Jang, Ph.D^1,2,3^, Sean Agbor-Enoh, MD^1,2,3,4^, Michael B. Keller, MD^1,2,3,4,6^

^1^Laborarory of Applied Precision Omics (APO) & ^2^Laboratory of Transplantation Genomics, National Heart, Lung and Blood Institute (NHLBI), National Institutes of Health, Bethesda, MD; ^3^Genomic Research Alliance for Transplantation (GRAfT), ^4^Pulmonary and Critical Care Medicine, Johns Hopkins Hospital, Baltimore MD, ^5^Division of Hospital Medicine, Johns Hopkins Bayview Medical Center, Baltimore, MD, ^6^Critical Care Medicine Department, Clinical Center, National Institutes of Health, Bethesda, MD, ^7^Inova Fairfax Hospital, Falls Church, VA, ^8^Division of Pulmonary and Critical Care Medicine, University of Maryland Medical Center, Baltimore MD, ^9^Genome Transplant Genomics (GTD), ^10^Division of Cardiovascular Medicine and ^11^Department of Pathology, Stanford University School of Medicine, Palo Alto, CA

**Supplementary Table 1: Treatment Practices for AMR and dnDSA without AMR at Participating Centers**

| Center | Treatment of AMR | Treatment of dnDSA without AMR |
| --- | --- | --- |
| Stanford | Plasmapheresis, High-dose methylprednisolone, 3-6 months of IVIG. Adjunct therapies: eculizumab, bortezomib, carfilzomib | No treatment |
| Johns Hopkins | Plasmapheresis, High-dose methylprednisolone, 3-6 months of IVIG. Adjunct therapies: eculizumab, bortezomib, carfilzomib | - Prior to 2016: No treatment  - After 2016: Rituximab and IVIG. For patients with concurrent infection, IVIG only |
| Inova Fairfax | Plasmapheresis, High-dose methylprednisolone, 3-6 months of IVIG. Adjunct therapies: eculizumab, bortezomib, carfilzomib | - Prior to 2017: No treatment  - After 2017: Bortezomib and IVIG. For patients with crossmatch level DSA, rituximab and IVIG |
| University of Maryland | Plasmapheresis, High-dose methylprednisolone, 3-6 months of IVIG. Adjunct therapies: eculizumab, bortezomib, carfilzomib | No treatment |

Abbreviations: AMR: Antibody-mediated rejection. dnDSA: de novo donor-specific antibodies. IVIG: intravenous immunoglobulin
